# An analysis of the role of GAB2 in pan-cancer from a multidimensional perspective

**DOI:** 10.1007/s12672-024-01135-5

**Published:** 2024-07-12

**Authors:** Yi Yin, Yong Li, Yaoyang Zhang, Qiucheng Jia, Huiming Tang, Jiming Chen, Rui Ji

**Affiliations:** 1 Department of Gynecology, Tumor Hospital Affiliated to Nantong University, Nantong, 226006 Jiangsu China; 2 Department of Obstetrics and Gynecology, The Second Affiliated Hospital of Dalian Medical University, Dalian, 116000 Liaoning China; 3 Department of Obstetrics and Gynecology, The Affiliated Changzhou Second People’s Hospital of Nanjing Medical University, Changzhou, 213003 Jiangsu China

**Keywords:** GAB2, Cancer, Prognosis, Phosphorylation, Immune infiltration

## Abstract

**Background:**

To explore the role of GAB2 in pan-cancer based on bioinformatics analysis.

**Methods:**

Based on TCGA and GTEx databases, we used TIMER2.0 online analysis tool and R language to analyze the expression of GAB2 in pan-cancer. We used Kaplan–Meier Plotter to analyze the relationship between GAB2 and OS and RFS in pan-cancer. We utilized the CPTAC database to examine the expression of phosphorylated GAB2 in pan-cancer. We investigated the effects of mutation features on the occurrence and development of human cancers by cBioPortal and COSMIC. Using the database, we conducted an analysis of molecular compounds that have the potential to interact with GAB2 through molecular docking. Moreover, we use the TIMER to explore the relationship between GAB2 and immune cell infiltration, and draw relevant heatmaps by R language.

**Results:**

GAB2 was abnormally expressed in various tumors and was associated with prognosis. There were differences in the expression of GAB2 phosphorylation in tumor tissues and corresponding normal tissues among different types of tumors. GAB2 interacts with Docetaxel and was associated with immune cell infiltration in various tumors.

**Conclusion:**

GAB2 participates in regulating immune infiltration and affects the prognosis of patients. GAB2 may serve as a potential tumor marker.

## Introduction

In many countries, more and more people are dying from cancer, which has become the first or second cause of death [1]. At present, the main treatment for cancer is still surgery, chemotherapy and radiotherapy. In recent years, immunotherapy, targeted therapy and endocrine therapy have gradually become the key treatments for cancer [2]. However, the survival rate of most cancer patients is still low [3]. Therefore, it is very necessary to investigate the potential therapeutic targets of cancer.

Grb2-associated binder family proteins (GABs) are a class of scaffold proteins, which widely express in the human body. Studies have confirmed that this family of proteins, including GAB1-4, Ne GAB, Soc1, and DOS, was an important element in the signaling pathway and a key regulatory factor and mediator in many biological physiological processes. GABs integrate and amplify stimulates from signaling molecules, including growth factors, cytokines, B/T antigen receptors, and cell adhesion molecules. It also promotes diversification of signal transduction by transmitting information from activated receptors to signaling pathways with different biological functions, playing an important role in various human diseases, especially malignant tumors [4–6]. GAB2, a major member, is mainly composed of a pleckstrin homology (PH) domain at the N-terminus, proline-rich motifs, and multiple tyrosine residues [7, 8]. PH domain can identify membrane component, especially phosphatidylinositol 3,4,5-triphosphate (PIP3), contributing to membrane recruitment. Proline-rich motifs contains PXXP domain structure, binding to the proteins which include SH3 domain. Multiple tyrosine residues can combine with with SH2 domain structure of the protein [9], such as SHP2, the Crk adaptor protein and the regulatory subunit of PI3K, p85 [5]. GAB2 phosphorylates under the stimulation of cytokines. The phosphorylated GAB2 combines with the p85 subunit of PI3K, and then plays a role in promoting cancer [10]. It has also been reported that phosphorylated GAB2 can also activate RAS/ERK signaling pathway and promote the occurrence and development of tumors [11]. Thus, the role of GAB2 in tumor is related to its phosphorylation. GAB2 has characteristics related to oncogenes and plays a significant role in the occurrence and development of various malignant tumors. Researches have shown that GAB2 highly expressed in breast cancer, colorectal cancer, bladder cancer, osteosarcoma, ovarian cancer and other tumors [12]. Horst et al. found that GAB2 promoted the proliferation, migration, and metastasis of melanoma cells in vivo by activating the PI3K/AKT signaling pathway [13]. GAB2 had abnormal expression in breast cancer and cell lines. Overexpression of GAB2 in human breast epithelial cell line (MCF10A) promoted cell proliferation [14].

In this study, we used data from The Cancer Genome Atlas (TCGA) and Genotype-Tissue Expression (GTEx) to investigate the expression of GAB2 and its relationship with prognosis. We also analyzed GAB2 mutations and copy number changes. Subsequently, we further analyzed the compounds that interact with GAB2 using molecular docking, and examined the relationship between GAB2 and immune infiltration. Our research findings suggest that GAB2 can serve as a potential tumor biomarker.

## Methods

### Gene expression analysis

We input GAB2 in Gene_DE module of Timer2.0 website to obtain the expression of GAB2 mRNA in pan-cancer in TCGA project. Based on TCGA and GTEx databases, we explored the expression of GAB2 in pan-cancer using the “ggplot2” package in R.Full names of various cancers were shown in Table 1.

**Table 1:** The abbreviations and corresponding full names of various cancers

Abbreviation	Full name
ACC	Adrenocortical carcinoma
BLCA	Bladder Urothelial Carcinoma
BRCA	Breast invasive carcinoma
CESC	Cervical squamous cell carcinoma and endocervical adenocarcinoma
CHOL	Cholangiocarcinoma
COAD	Colon adenocarcinoma
DLBC	Lymphoid Neoplasm Diffuse Large B-cell Lymphoma
ESCA	Esophageal carcinoma
GBM	Glioblastoma multiforme
HNSC	Head and Neck squamous cell carcinoma
KICH	Kidney Chromophobe
KIRC	Kidney renal clear cell carcinoma
KIRP	Kidney renal papillary cell carcinoma
LAML	Acute Myeloid Leukemia
LGG	Brain Lower Grade Glioma
LIHC	Liver hepatocellular carcinoma
LUAD	Lung adenocarcinoma
LUSC	Lung squamous cell carcinoma
MESO	Mesothelioma
OV	Ovarian serous cystadenocarcinoma
PAAD	Pancreatic adenocarcinoma
PCPG	Pheochromocytoma and Paraganglioma
PRAD	Prostate adenocarcinoma
READ	Rectum adenocarcinoma
SARC	Sarcoma
SKCM	Skin Cutaneous Melanoma
STAD	Stomach adenocarcinoma
TGCT	Testicular Germ Cell Tumors
THCA	Thyroid carcinoma
THYM	Thymoma
UCEC	Uterine Corpus Endometrial Carcinoma
UCS	Uterine Carcinosarcoma
UVM	Uveal Melanoma

### Survival prognosis analysis

Kaplan–Meier Plotter was used in analyzing the correlation between GAB2 mRNA expression and Overall Survival (OS) and Relapse free survival (RFS).

### Phosphorylation expression analysis

The expression level of GAB2 phosphorylation was explored by using the ualcan portal. We examined the expression level of the phosphoprotein of GAB2 in both primary tumor and normal tissues, respectively, by entering “GAB2”. The available datasets of six tumors were selected.

### Mutation status analysis

“Pan-cancer analysis of whole genomes (ICGC/TCGA, Nature 2020)” section of cBioPortal was used in exploring the genetic alteration status of GAB2. The ‘Cancer Types Summary’ module was used to analyze the overview of GAB2 mutation status and the ‘Mutations’ module was used to show GAB2 mutation sites and corresponding 3D structures. Moreover, the ‘Comparison/Survival’ module was used to explore the influence of GAB2 mutation status on pan-cancer survival prognosis. COSMIC was applied to verify the pan-cancer GAB2 mutation features. We used GSCA to analyze the correlation between GAB2 expression and CNV in different cancers and the effect of GAB2 CNV on the pan-cancer survival prognosis.

### Molecular docking

First, we predicted the molecule compound acting on GAB2 in GSCA database, found the 3D structure information of the molecule compound in PubChem database, and downloaded the GAB2 protein structure through PDB database. Then, we used Discovery Studio software to GAB2 and molecules of the ingredients were processed, and docking started after processing.

### Immune cell infiltration

In the GENE module of the TIMER database, enter “GAB2” in Gene Symbol, select all tumor types in Cancer Types, and select “B cell, CD8+ Tcell, CD4+ T cell, Macrophage, Neutrophil, Dendritic Cell” in Immune Infiltrates, Submit analysis. Download the p value file. Organize the p-value and partial.cor values in the p-value file into an immune infiltration analysis file. Visualize data and draw correlation heatmaps using the “ggplot2” package of R.

## Result

### Expression level of GAB2 mRNA in pan-cancer

**Fig. 1:**
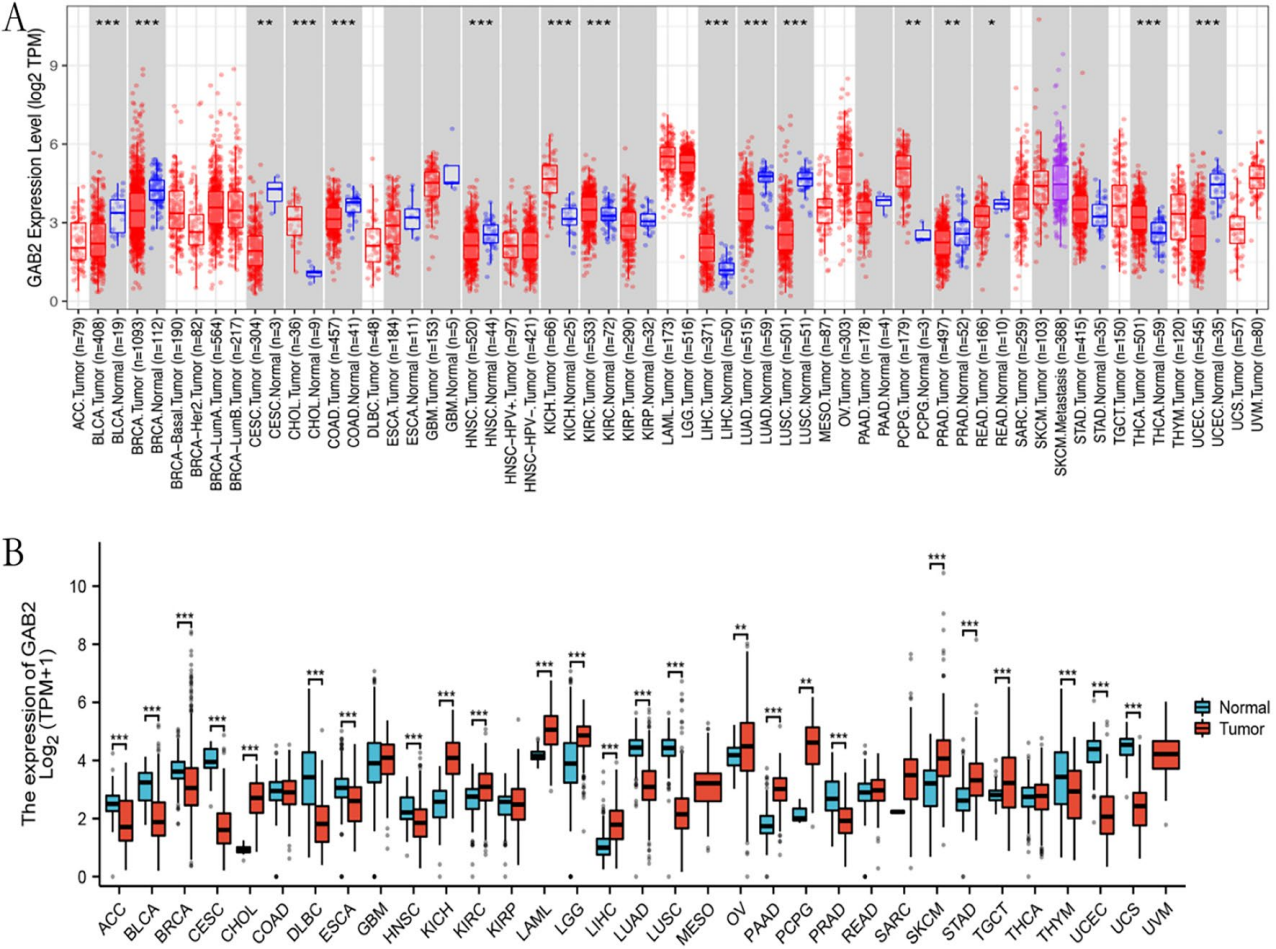
GAB2 mRNA expression levels in cancers. **A** GAB2 mRNA expression levels in pan-cancer from TCGA data in TIMER 2.0. **B** GAB2 mRNA expression levels in pan-cancer from TCGA and GTEx data. *P < 0.05, **P < 0.01, ***P < 0.001

We utilized the TIMER 2.0 online tool to explore the expression of GAB2 mRNA in tumor tissues and corresponding normal tissues in the TCGA database. As shown in Fig. 1A, the expression level of GAB2 mRNA increased in cancer in CHOL, KICH, KIRC, LIHC, PCPG, and THCA, while decreased in cancer in BLCA, BRCA, CESC, COAD, HNSC, LUAD, LUSC, PRAD, READ, and UCEC.

Due to the little or no normal samples of some tumors in the TCGA database, we conducted further analysis in TCGA and GTEx database. As shown in Fig. 1B, the expression level of GAB2 mRNA increased in some tumors, such as CHOL, KICH, KIRC, LAML, LGG, LIHC, OV, PAAD, PCPG, SKCM, STAD, and TGCT; and the expression was reduced in cancer in ACC, BLCA, BRCA, CESC, DLBC, ESCA, HNSC, LUAD, LUSC, PRAD, THYM, UCEC, and UCS.

### The relationship between GAB2 and prognosis in cancer

**Fig. 2:**
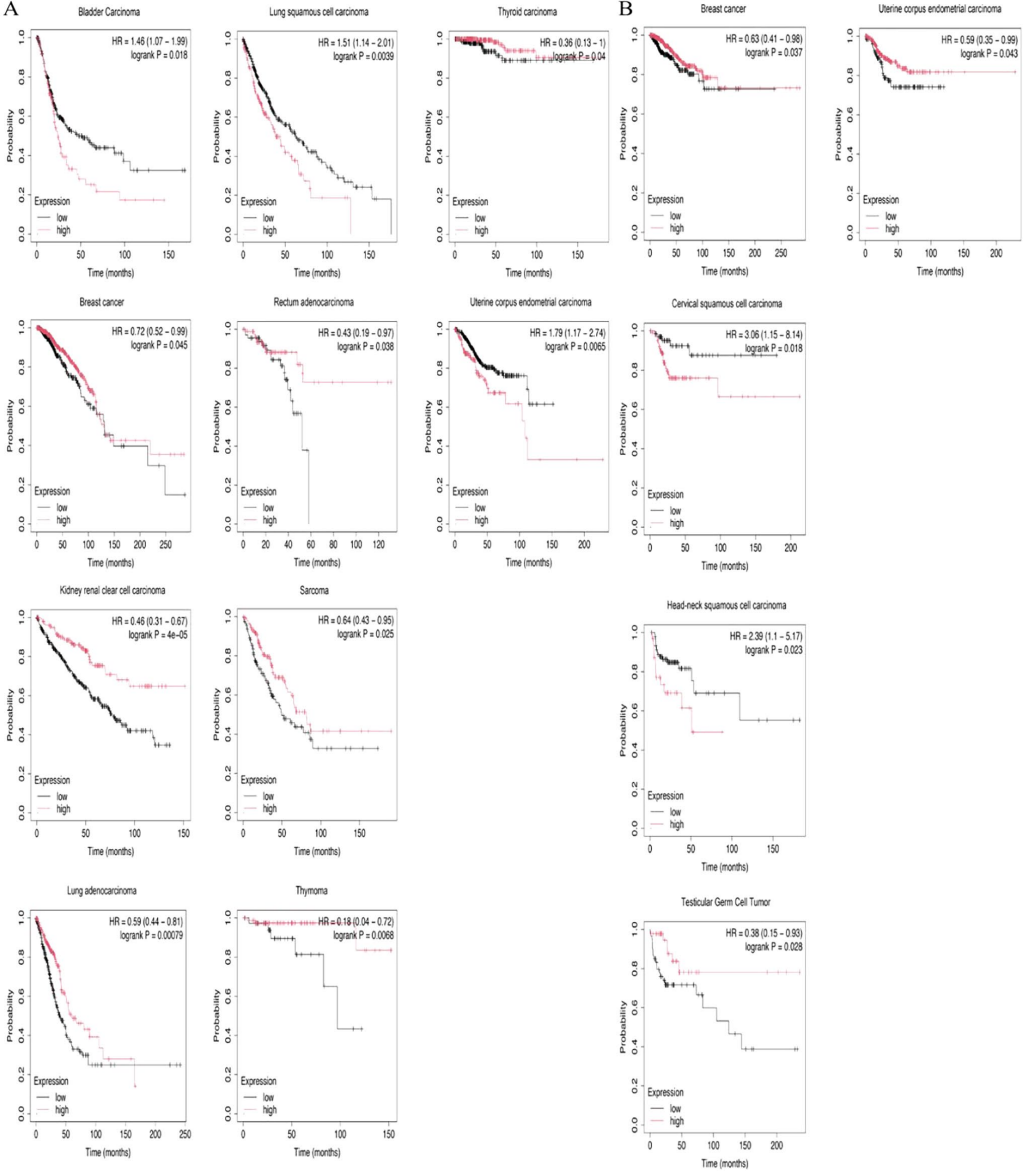
Survival analysis of GAB2 in pan-cancer. **A**)Correlation of GAB2 expression with OS in patients. **B** Correlation of GAB2 expression with RFS in patients

To further clarify the impact of GAB2 expression on the prognosis of patients. Based on the TCGA database, we used Kaplan–Meier Plotter to analyze the relationship between GAB2 expression and OS and RFS in pan-cancer. The result was shown in Fig. 2A, in BLCA, LUSC, and UCEC, high levels of GAB2 indicated shorter OS. However, we still found the opposite phenomenon, patients with high GAB2 expression had significantly longer OS in BRCA, KIRC, LUAD, READ, SARC, THYM and THCA. Subsequently, we further analyzed the predictive significance of GAB2 for RFS (Fig. 2B). It was found that high level of GAB2 was associated with shorter RFS in certain tumors, such as CESC and HNSC. In BRCA, TGCT and UCEC, the high level of GAB2 was associated with longer RFS.

### Phosphorylation analysis of GAB2 in pan-cancer

**Fig. 3:**
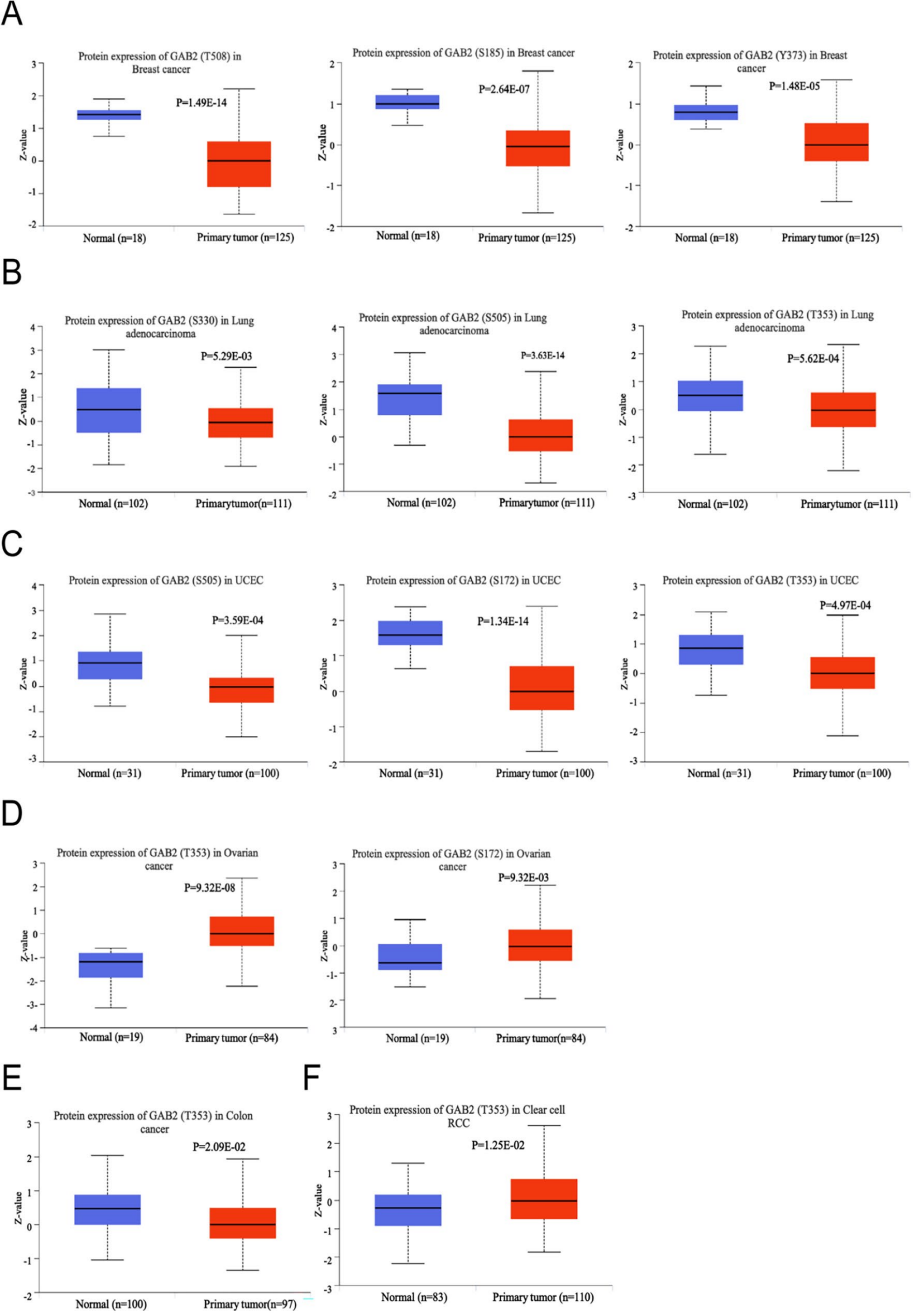
Phosphorylation analysis of GAB2 protein in different tumors. Based on the CPTAC dataset, we analyzed the expression level of GAB2 phosphoprotein between normal tissue and tumor tissue. We supply the box plots for different cancers, including **A** breast cancer, **B** Lung adenocarcinoma, **C** UCEC, **D** Ovarian cancer, **E** Colon cancer, **F** clear cancer RCC. Product-limit method was used

As shown in Fig. 3, in breast cancer, the GAB2 phosphorylation of T508, S185 and Y373 sites is higher in normal tissue. GAB2 phosphorylation of S330, S505, and T353 shows higher expression in normal tissue in Lung adenocarcinoma. GAB2 phosphorylation of S505, S172, and T353 in normal tissue is higher than that in primary tumor tissue in UCEC. GAB2 phosphorylation of T353 shows higher expression in normal tissue in colon cancer. However, in ovarian cancer, GAB2 phosphorylation of T353 and S172 in tumor tissues were significantly higher than those in normal tissues. Meanwhile, GAB2 phosphorylation of T353 was also higher in the tumor in renal clear cell carcinoma.

### Analysis of GAB2 mutation in pan-cancer

**Fig. 4:**
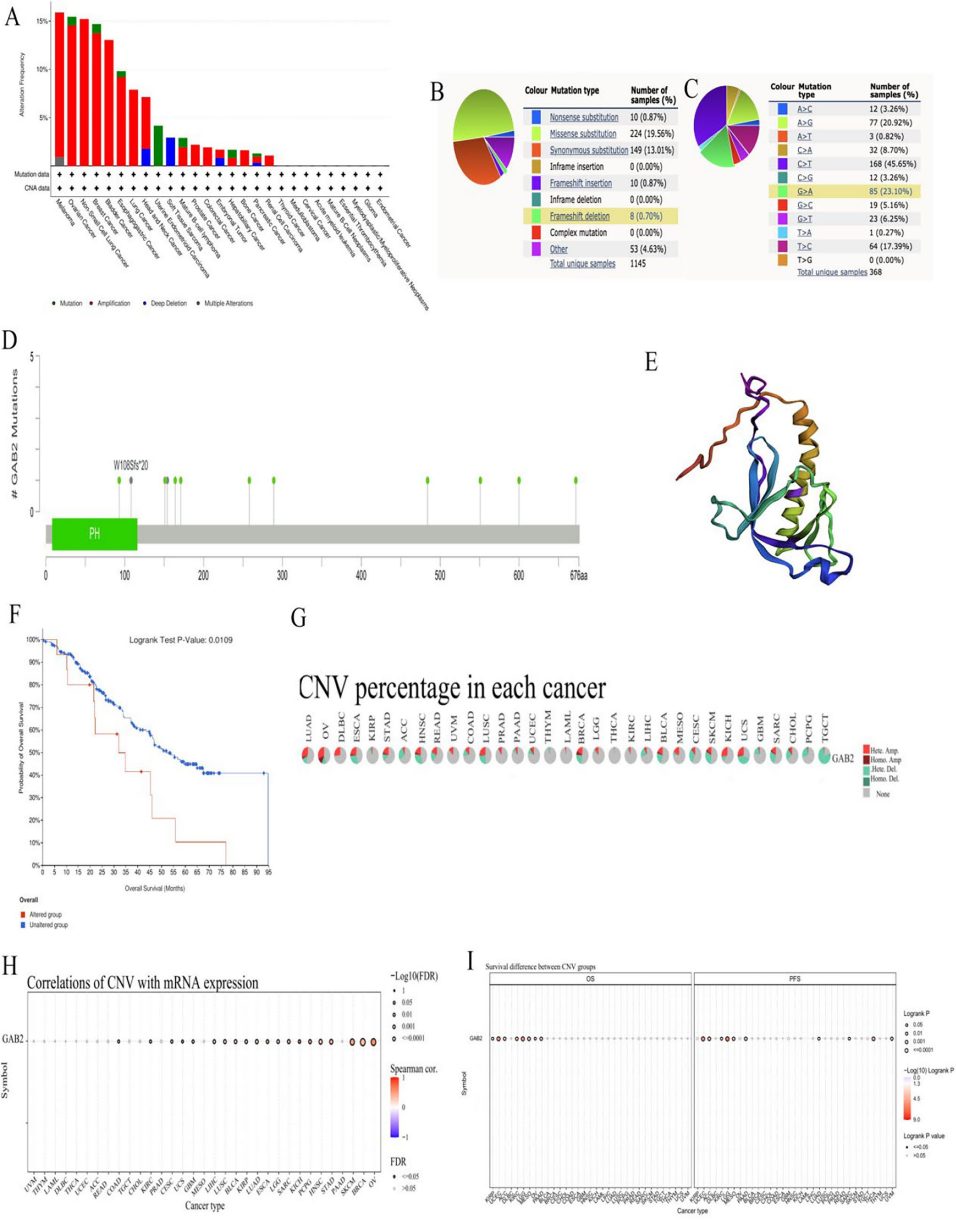
Mutation features of GAB2 in human cancers. Overview of the mutation status of GAB2 across TCGA cancers by **A** cBioPortal and **B** COSMIC. **C** Summary of GAB2 substitutional mutation types by COSMIC. **D** Mutation sites and **E** corresponding 3D structures of GAB2 displayed by cBioPortal. The correlations between pan-cancer GAB2 mutation status and **F** OS. **G** Summary of GAB2’s CNV across TCGA cancers. **H** The correlation between GAB2 expression and CNV in different cancers by GSCA. **I** The difference of OS and PFS between GAB2’s CNV and wide type in different cancers by GSCA

We used cBioPortal and COSMIC to explore GAB2 mutative status. As shown in Fig. 4A, the highest GAB2 alteration frequency occurred in melanoma, followed by OV, NSCLC, BRCA and BLCA. A variety of cancers were discovered to have GAB2 amplification alteration. From the GAB2 mutation sites (Fig. 4D) and corresponding 3D structures (Fig. 4E) W108Sfs*20/Pfs*20 was the most frequent mutation site. GAB2 mutation sites and their corresponding 3D structures were shown in Fig. 4D. Missense substitutions were the major mutation type and took 19.56% of 1145 GAB2 mutation (Fig. 4B). Moreover, GAB2 substitution mutations mostly happened on C>T (45.65%) and G>A (23.10%) (Fig. 4C). In addition, we utilized cBioPortal to examine the impact of GAB2 alterations on OS across multiple cancer types. The results showed that GAB2 alterations could significantly shorten OS (p = 0.0109, Fig. 4F). Then, we explored the distribution of different types of GAB2’s CNV (Fig. 4G) and the correlations between GAB2 expression and CNV (Fig. 4H). We found that The significant correlations between GAB2 expression and CNV could be found in COAD, KIRC, CESC, UCS, GBM, LIHC, LUSC, BLCA, KIRP, LUAD, ESCA, LGG, SARC, KICH, PCPG, HNSC, STAD, SKCM, BRCA and OV. Furthermore, the CNV of GAB2 showed a strong association with unfavorable OS in various cancer including KIRP, UCEC, ACC, KIRC, LGG, MESO, OV, and PAAD as well as poor PFS in 10 cancer types (UCEC, ACC, KIRC, LGG,MESO, PAAD, LUAD, SARC THCA and UCS) (Fig 4I).

### Molecular docking

**Fig. 5:**
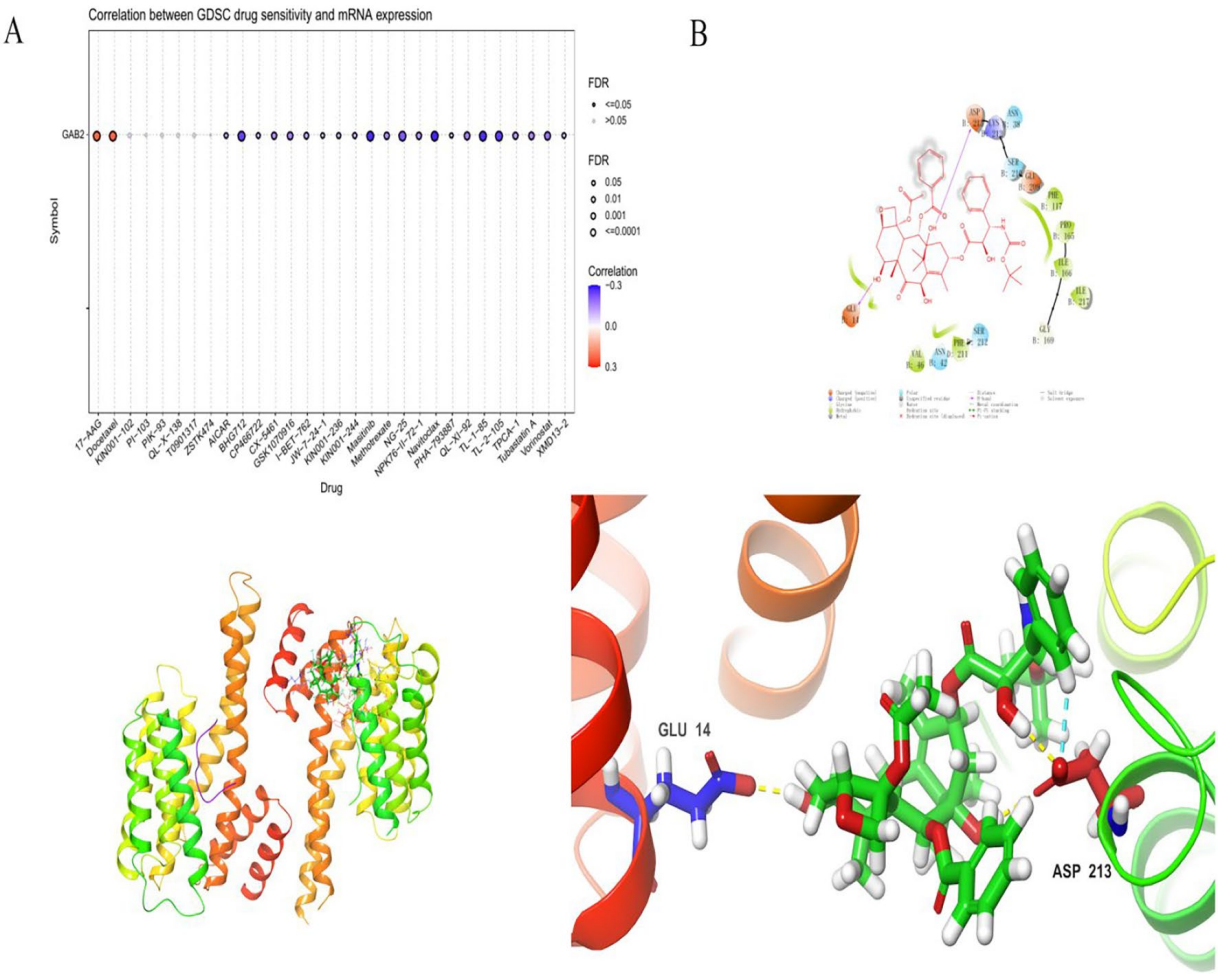
Molecular docking. **A** Main molecular compounds acting on GAB2. **B** Molecular docking results of docetaxel and GAB2

Use GSCA database to screen out molecular compounds acting on GAB2, and the results are shown in the Fig. 5A. In the screening results, we found that Docetaxel can act on GAB2, and the correlation is relatively large (Fig. 5B). Docetaxel is also a commonly used clinical anti-tumor drug, so we chose Docetaxel for further analysis. We docked Docetaxel with GAB2 and analyzed the binding energy obtained. The binding energy is negative. The larger the result, the lower the energy, and the more stable the binding between target and molecule. The final binding energy of Docetaxel and GAB2 was − 4.875. This shows that Docetaxel and GAB2 combined stably.

### Performing correlation analysis between GAB2 expression and the presence of infiltrating immune cells

**Fig. 6:**
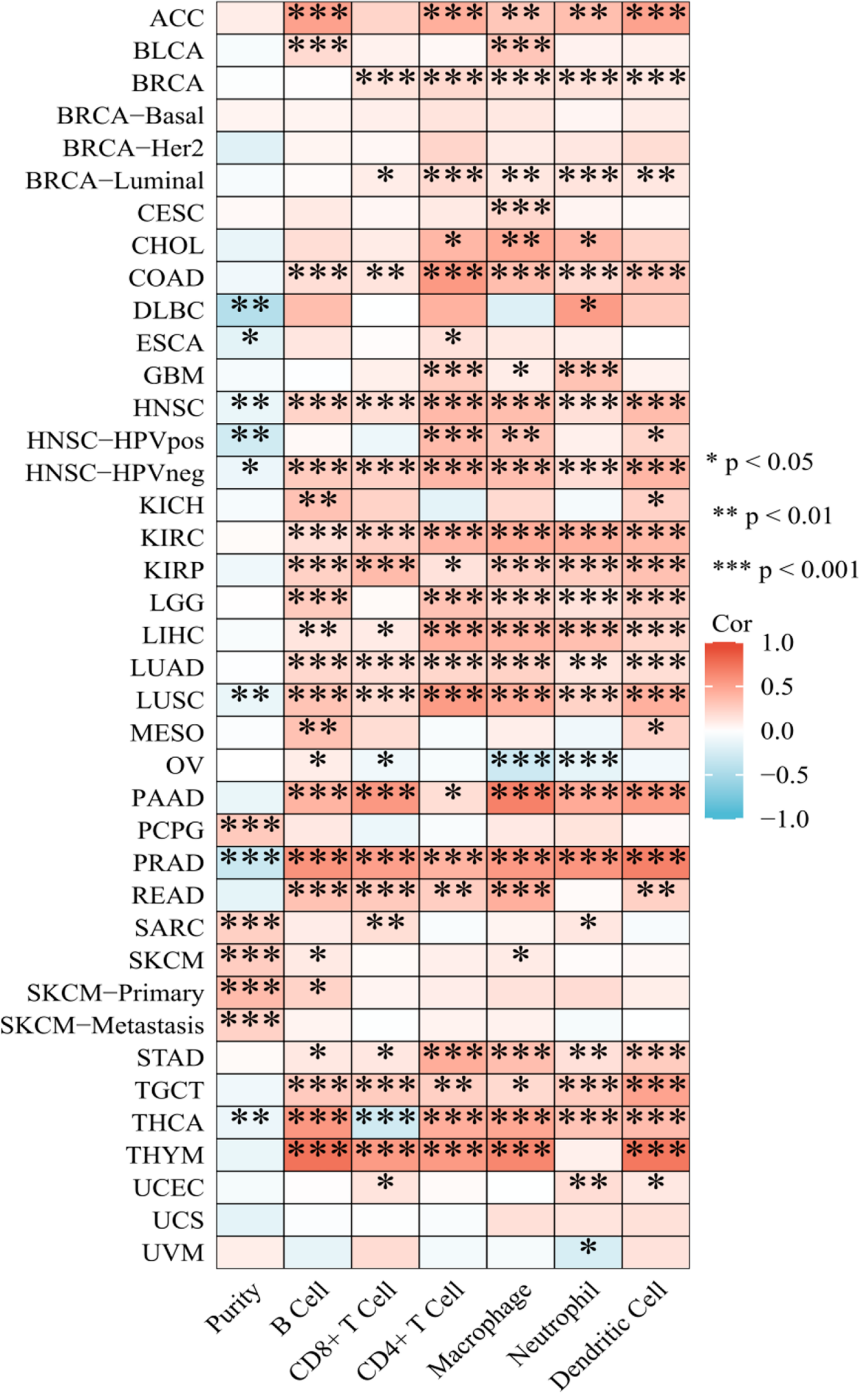
Correlation of GAB2 expression with immune infiltration

To explore the correlation between GAB2 and the degree of immune cell infiltration. We used the TIMER database to analyze the relationship between GAB2 and the degree of infiltration of B cells, CD4+ T cells, CD8+ T cells, neutrophils, macrophages, and dendritic cells in pan-cancer. We found immune cell infiltration in various tumors, such as ACC, BRCA, COAD, HNSC, KIRC, KIRP, LGG, LIHC, LUAD, LUSC, PAAD, PRAD, READ, STAD, TGCT, THCA, and THYM (Fig. 6).

## Discussion

As a type of connexin, GABs family proteins are involved in various intracellular signal transduction and are key molecules in downstream signal transduction pathways activated by tyrosine kinases (PTKs). GAB2 recruits signal transporters rich in the SH2 domain, to activate downstream signal transduction pathways such as PI3K/AKT and SHP2/Ras/ERK, when GAB2 is phosphorylated by PTKs. GAB2 plays an important role in physiological processes such as cell proliferation, differentiation, and migration [15]. Recent studies have found that GAB2 was highly expressed in breast cancer, ovarian cancer, lung cancer, glioma and other malignant tumors, and was involved in regulating the occurrence and metastasis of tumors [16–20]. In this study, we conducted a comprehensive bioinformatics analysis of GAB2 using multiple public databases.

Our research results show that based on the TCGA database or the TCGA database and GTEx database, the expression level of GAB2 mRNA is increased in CHOL, KICH, KIRC, LIHC, and PCPG. Our research results are not completely consistent with previous reports, which may be related to the little samples or no normal samples. In addition, this study mainly compared the expression differences of GAB2 mRNA between tumor and corresponding normal tissue, and previous reports have mostly compared the expression differences of GAB2 at the protein level. Subsequently, we further explored its prognostic significance in pan-cancer. The results showed that in BLCA, LUSC, and UCEC, high levels of GAB2 indicated shorter OS. However, we still found the opposite phenomenon, patients with high GAB2 expression had significantly longer OS in BRCA, KIRC, LUAD, READ, SARC, THYM and THCA. In summary, the expression level of GAB2 mRNA has a significant impact on the prognosis of patients in various types of cancer. Similarly, previous studies have indicated that patients with high GAB2 mRNA expression in LUSC have significantly shorter overall survival, while the opposite is observed in KIRC [21]. Other studies have shown that patients with high GAB2 mRNA expression in READ have a better prognosis [22]. These differences may be related to the biological functions and mechanisms of GAB2 in different cancers.

In addition, we also found that high levels of GAB2 mRNA are associated with shorter RFS in CESC and HNSC. In BRCA, TGCT and UCEC, the high level of GAB2 was associated with longer RFS. Therefore, we guess that GAB2 can serve as a potential biomarker for predicting the prognosis of patients.

After being phosphorylated by tyrosine kinase, GAB2 activates downstream signal transduction pathways, playing an important role in cell proliferation, differentiation, invasion, and metastasis [23]. S159 in GAB2 is a negative regulatory site. After phosphorylation, S159 can act on the AKT signaling pathway [24]. S210 and T391 are 14-3-3 binding and negative regulatory sites [25, 26]. Y584 can bind to the p85 subunit of PI3K after phosphorylation [7]. In this study, we found that the phosphorylation level at S505 differed in lung adenocarcinoma, ovarian cancer, and UCEC. In contrast, the phosphorylation level at T353 exhibited disparities between ovarian cancer and renal clear cell carcinoma. The phosphorylation of tyrosine residues T353 and S505 may have a significant impact on multiple phosphorylation sites. In this study, we discovered a positive correlation between Docetaxel and GAB2, suggesting that Docetaxel has the ability to stimulate the expression of GAB2. We also found that Docetaxel can bind to GAB2 stably. Our research and previous reports found that GAB2 is highly expressed in ovarian cancer, gastric cancer, and prostate cancer. In clinical treatment, Docetaxel is often used to treat non-small cell lung cancer, ovarian cancer, prostate cancer, gastric cancer, breast cancer and other malignant tumors, and has achieved significant therapeutic effects. However, it is still unclear whether Docetaxel exerts anti-tumor effects by acting on GAB2, and further verification is needed.

It has been reported that the development of GAB2-induced colitis in mice is associated with increased T cell invasion [27]. GAB2 could potentially be associated with immune system activation. In our study, we found that GAB2 was associated with immune cell infiltration in ACC, BRCA, COAD, HNSC, KIRC, KIRP, LGG, LIHC, LUAD, LUSC, PAAD, PRAD, READ, STAD, TGCT, THCA and THYM. Zhou et al. found that SNORA38B reshapes the tumor microenvironment by regulating the GAB2/AKT/mTOR signaling pathway, reducing tumorigenesis in non-small cell lung cancer and enhancing immune checkpoint blockade [28]. There is limited research on the relationship between GAB2 and immune cell infiltration and the efficacy of immunotherapy. Further experiments are needed to verify the impact of GAB2 on the efficacy of immunotherapy.

Overall, we analyzed GAB2 using bioinformatics methods, and the results showed that GAB2 was abnormally expressed in various tumors and was associated with prognosis. We also found that GAB2 interacts with Docetaxel and was associated with immune cell infiltration in various tumors. It provides new possible ideas for studying the role of GAB2 in tumor occurrence and development.

## Limitation

This study has several limitations: first, it relies on online databases, online tools, and the R language to process data from multiple databases, including TCGA, GTEx, and others, which may introduce errors in the statistical methods. Second, there is a lack of additional clinical and experimental data to further validate the expression, function, and prognostic significance of GAB2 in pan-cancer.

## Data Availability

Publicly available datasets were analyzed in this study. This data can be found here: https://cistrome.shinyapps.io/timer/, http://gepia.cancer-pku.cn/, http://kmplot.com/analysis/index.php?p=background, http://ualcan.path.uab.edu/, http://geneontology.org/, http://www.cbioportal.org/, https://cancer.sanger.ac.uk/cosmic, https://pubchem.ncbi.nlm.nih.gov/.
